# Mandibular Jaw Movement and Masticatory Muscle Activity during Dynamic Trunk Exercise

**DOI:** 10.3390/dj8040132

**Published:** 2020-12-02

**Authors:** Daisuke Sugihara, Misao Kawara, Hiroshi Suzuki, Takashi Asano, Akihiro Yasuda, Hiroki Takeuchi, Toshiyuki Nakayama, Toshikazu Kuroki, Osamu Komiyama

**Affiliations:** Division of Oral Function and Rehabilitation, Department of Oral Health Science, Nihon University School of Dentistry at Matsudo, 870-1 Sakaecho, Nishi-2, Matsudo, Chiba 271-8587, Japan; mada16015@g.nihon-u.ac.jp (D.S.); kawara.misao@nihon-u.ac.jp (M.K.); asano.takashi@nihon-u.ac.jp (T.A.); yasuda.akihiro@nihon-u.ac.jp (A.Y.); hiroki.snowdon.takeuchi@gmail.com (H.T.); mato19015@g.nihon-u.ac.jp (T.N.); kuroki.toshikazu@nihon-u.ac.jp (T.K.); komiyama.osamu@nihon-u.ac.jp (O.K.)

**Keywords:** mandibular jaw movement, masticatory muscle activity, dynamic trunk exercise, mandibular fixation, bracing

## Abstract

The examination of jaw movement during exercise is essential for an improved understanding of jaw function. Currently, there is no unified view of the mechanism by which the mandible is fixed during physical exercise. We hypothesized that during strong skeletal muscle force exertion in dynamic exercises, the mandible is displaced to a position other than the maximal intercuspal position and that mouth-opening and mouth-closing muscles simultaneously contract to fix the displaced mandible. Therefore, we simultaneously recorded mandibular jaw movements and masticatory muscle activities during dynamic trunk muscle force exertion (deadlift exercise) in 24 healthy adult males (age, 27.3 ± 2.58 years). The deadlift was divided into three steps: Ready (reference), Pull, and Down. During Pull, the mandibular incisal point moved significantly posteriorly (−0.24 mm, *p* = 0.023) and inferiorly (−0.55 mm, *p* = 0.019) from the maximal intercuspal position. Additionally, temporal, masseter, and digastric muscles were activated simultaneously and significantly during Pull (18.63 ± 17.13%, 21.21 ± 18.73%, 21.82 ± 19.97% of the maximum voluntary contraction, respectively), with maintained activities during Down (*p* < 0.001). Thus, during dynamic trunk muscle force exertion, the mandibular incisal point moved to a posteroinferior position without tooth-touch (an open-mouth position). Simultaneously, the activities of the mouth-opening digastric muscles and the mouth-closing temporal and masseter muscles led to mandibular fixation, which is a type of mandible fixing called bracing.

## 1. Introduction

In everyday life, the mandible moves in accordance with the situation, such as mastication, swallowing, and phonation. The mandible is also known to move simultaneously with the head movements during full-body exercise. For example, during walking and running, the head oscillates vertically and may undergo rotational yaw and pitch. The velocity and frequency of head movements during such activities [1,2] and the nature of stabilizing head movements to maintain the constancy of gaze and posture have been described [3,4,5]. The vertical positional relationship of the mandible relative to the maxilla remains constant when such movements are not conducted; this condition is known as the mandibular rest position [6]. When examining mandibular dynamics during physical exercise using this scheme, it is hard to consider the mandible as placed in the mandibular rest or other relaxed positions. During physical exercise, the mandible should be fixed in a position that is optimal for the exercise. However, there is no unified view of the mechanism by which the mandible is fixed, and a search of the literature did not reveal any reports of natural jaw movements during exercise. However, examination of jaw movement during exercise is important for improved understanding of jaw function and for correct selection of materials and better occlusal adjustment during prosthetic treatment.

When humans exert whole-body muscle force during physical exercise, the mandible is frequently observed, in not only top athletes, but also sports lovers, to be fixed in a position other than the maximal intercuspal position, including open-mouth, protruded, and lateral positions [7]. This phenomenon is considered to occur because postural reflexes and a strong bite in the maximal intercuspal position reduce the reciprocal innervation involved in skeletal muscle contractions [8,9,10]. Therefore, a strong bite is considered to be effective during static exercise (i.e., during performance of an exercise with almost no movement, typified by grip strength exertion [11,12,13]) but has a negative effect during dynamic exercise [14,15,16,17]. Additionally, in terms of the mandibular position during exercise, the condylar point has a tendency to be displaced in the posterior and inferior directions of the sagittal plane during strong force exertion by the back muscles, with the digastric muscles (Dm) simultaneously and forcefully contracting [18,19,20]. Thus, masticatory muscle activities under muscle exertion during exercise with the trunk position maintained can cause various changes in other muscle activities. However, many previous investigations of mandibular dynamics focused on muscle force exertion during exercise with the mandible clenched in the maximal intercuspal position [11,12], or on masticatory muscle activities under muscle exertion during exercise [19,20]. Thus, the natural mandibular dynamics during muscle force exertion in humans have not yet been clarified; this knowledge gap should be addressed.

Therefore, we hypothesized that during strong skeletal muscle force exertion in dynamic exercise, the mandible is displaced to a position other than the maximal intercuspal position, and that mouth-opening and mouth-closing muscles simultaneously contract to fix the displaced mandible. To test this hypothesis, we simultaneously recorded and examined mandibular jaw movements and masticatory muscle activities during dynamic trunk muscle force exertion.

## 2. Materials and Methods

### 2.1. Participants

Twenty-four healthy adult males (age, 27.3 ± 2.58 years) who enjoyed sports in their everyday life and had a stable maximal intercuspal position, without malocclusion, toothache, orofacial myalgia, or temporomandibular joint arthralgia, were selected as participants. The selected participants also had a favorable general health status without medical history of injury. Each participant was preliminarily provided with adequate explanation of the objectives and methods of this study. Verbal and written informed consent was obtained from all participants prior to starting the experiment. This study was approved by the Ethics Committee at the Nihon University School of Dentistry at Matsudo (EC16-013).

### 2.2. Test Exercise

The deadlift, a powerlifting performance exercise in which the back is straightened, with the trunk posture maintained, was selected as the test exercise for dynamic trunk muscle force exertion. In this study, the course of the deadlift was divided into three steps. The first step comprised the movements in getting ready to lift, in which participants stood with their feet shoulder-width apart behind a barbell, bent their knees, and grabbed the bar (Ready). The second step comprised the movements in lifting the barbell along the front of the body, with force, while raising their body (Pull). The third step comprised the movements in putting the lifted barbell back into place (Down). This sequence of movements was defined as one attempt. During each attempt, the participants faced forward, with the bite plane parallel to the floor, according to the basic posture of the deadlift, and were instructed to hold this posture.

Prior to assessments, the participants performed adequate stretching and warm-up exercises for barbell lifting. After adequate rest, the participants completed three attempts. The barbell weight during the attempts was approximately 80% of the maximum weight the participant could lift, in consideration of their safety.

### 2.3. Jaw Movement Measurements

**Figure 1:**
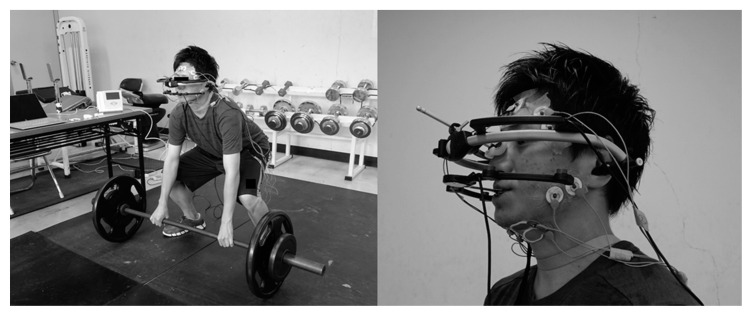
Measurement of mandibular jaw movement and electromyographic activity of the masticatory muscles during deadlift.

Jaw movements were recorded using a three-dimensional six-degree-of-freedom jaw movement measurement device (ARCUS digma2, KaVo, Biberach, Germany). The maxillary attachment (face-bow) of the ARCUS digma2 device was affixed using the Frankfort plane (FH) as a reference plane. A mandibular attachment (clutch) was made using a quick-cure resin (UNIFAST III, GC, Tokyo, Japan) and was fixed/bonded with a cyanoacrylate instant adhesive (Aron Alpha, Toagosei Co., Ltd.; Tokyo, Japan) while adjusting its position to prevent the maxillary teeth from touching the bilateral first molar teeth with the mandible in the maximal intercuspal position [21]. The ARCUS digma2 device was then affixed parallel to Camper’s plane by marking the middle and posterior ear canals and the subnasale (Figure 1).

Initially, the maxillary tooth alignment position with respect to the maxillary sensor and, subsequently, the mandible tooth alignment position with respect to the maxillary tooth alignment position in the maximal intercuspal position were recorded using the ARCUS digma2 transmitter (bite-fork). Anterior and right/left lateral movements were recorded using the articulator mode of the device. Subsequently, incisal point movements in the sagittal plane during the test exercise were measured using the analysis mode. At the start of each assessment, the mandible was placed in the maximal intercuspal position with a minimal bite force; this position was defined as the measurement origin. The participants then moved their jaw at their discretion, without any instructions. The maximum incisal point displacements in the anterior–posterior and superior–inferior directions of the sagittal plane were assessed during Ready, Pull, and Down.

### 2.4. Masticatory Muscle Activity Measurements

The assessment of masticatory muscle activities focused on the anterior bundles of the right and left temporal muscles (Tm) and the center of the superficial layer of the masseter muscles (Mm) (i.e., mouth-closing muscles), and the suprahyoid muscles, mainly the Dm muscles (i.e., mouth-opening muscles). Electrodes were affixed along the muscle fibers 10 mm posterior and parallel to the anterior margin of the Tm, at the center of and parallel to the anterior margin of the Mm, and 20 mm away from the chin and on the bisector of the angle between the line connecting the chin with the mandibular angle and the sagittal line of the chin (Dm) (Figure 1).

Masticatory contractions were measured using a multi-telemeter system (Polymate Mini AP108, Miyuki Giken Co., Ltd.; Tokyo, Japan) with an Ag-AgCl bipolar surface electrode (5 mm diameter). The derived electromyographic signals were scanned by the wave-analyzing software, BIMUTAS-Video (KISSEI COMTEC Co., Ltd.; Matsumoto, Japan), and recorded into a personal computer with a sampling frequency of 1 kHz. Measurements were taken with the following settings: with the treble cut-off frequency turned off, a time constant of 0.03 s, and a sensitivity (SENS) of 0.5 mV/diV. In addition, the contractions of the Tm and Mm during maximum voluntary bite force exertion in the maximal intercuspal position, and that of the Dm under maximum voluntary resistance against mouth-opening (achieved by placing the thumbs on the lower margin of the mandibular midline to prevent the head from bending backward) were measured to define the maximum voluntary contractions (MVCs) of the Tm, Mm, and Dm, respectively.

In the analysis of electromyographic data, the mean contraction in 1 s of stable electromyographic waveforms out of 3 s of electromyographic waveforms obtained at the time of the maximum voluntary bite (Tm and Mm) or the maximum voluntary resistance against mouth-opening (Dm) was taken as the MVC. For each step of the deadlift (Ready, Pull, Down), 0.2 s of stable electromyographic waveforms were selected, and the percentages of the Tm, Mm, and Dm relative to the corresponding MVC (root mean square (RMS)) were calculated. Additionally, jaw movement measurements and electromyography were simultaneously performed, and for all analyses, the barbell position was confirmed using a video camera.

### 2.5. Statistical Analyses

The maximum incisal point displacements in the anterior–posterior and superior–inferior directions of the sagittal plane, during the three steps of the deadlift (Ready, Pull, Down) were examined using a repeated-measures analysis of variance (ANOVA) with step as a factor. The activities of Tm, Mm, and Dm during the three steps of the deadlift (Ready, Pull, Down) were also examined using a repeated-measures ANOVA with step as a factor. For both analyses, the least significant difference method was used in subsequent multiple comparisons. Analyses were performed using SPSS version 24 software (IBM SPSS Japan Inc.; Tokyo, Japan). Statistical significance was set at 5%.

## 3. Results

### 3.1. Mandibular Incisal Point Displacements

During the deadlift, the incisal point displacement from Ready (baseline: 0.00 ± 0.00 mm) in the anterior–posterior direction of the sagittal plane was −0.24 ± 0.50 mm during Pull and −0.17 ± 0.35 mm during Down. The ANOVA revealed that the step factor was significant (F(2, 46) = 4.12, *p* = 0.023), and the subsequent multiple comparisons revealed significant differences in the mean displacement between Ready and Pull (*p* = 0.028) and between Ready and Down (*p* = 0.030). Specifically, the incisal point was displaced posteriorly from the maximal intercuspal position during Pull, which was maintained during Down. Additionally, the incisal point displacement from Ready (baseline: 0.00 ± 0.00 mm) in the superior–inferior direction of the sagittal plane was −0.55 ± 1.07 mm during Pull and −0.30 ± 0.97 during Down. The ANOVA revealed that the step factor was significant (F(2, 46) = 4.30, *p* = 0.019), and the subsequent multiple comparisons revealed a significant difference in the mean displacement between Ready and Pull (*p* = 0.019). Specifically, the incisal point was displaced inferiorly from the maximal intercuspal position to an open-mouth position during Pull (Table 1).

### 3.2. Masticatory Muscle Activities

The Tm contraction was 6.90 ± 4.19% of the MVC during Ready, 18.63 ± 17.13% during Pull and 10.39 ± 12.11% during Down. The ANOVA revealed that the step factor was significant (F(2, 46) = 11.44, *p* < 0.001), and the subsequent multiple comparisons revealed significant differences in the mean Tm contraction between Ready and Pull and between Pull and Down (both *p* < 0.001). The Tm contraction increased during Pull and then decreased during Down, to the same value as that during Ready.

The Mm contraction was 7.06 ± 5.00% of the MVC during Ready, 21.21 ± 18.73% during Pull and 11.79 ± 11.94% during Keep. The ANOVA revealed that the step factor was significant (F(2, 46) = 18.13, *p* < 0.001), and the subsequent multiple comparisons revealed significant differences in the mean Mm contraction among all steps (all *p* < 0.05). Specifically, the Mm contraction increased during Pull, and then decreased during Down. The Dm contraction was 6.98 ± 4.25% of the MVC during Ready, 21.82 ± 19.97% during Pull, and 13.82 ± 14.43% during Down. The ANOVA revealed that the step factor was significant (F(2, 46) = 15.56, *p* < 0.001), and the subsequent multiple comparisons revealed significant differences in the mean Dm contraction among all steps (all *p* < 0.05). Specifically, the Dm contraction increased during Pull, and then decreased during Down (Table 2).

## 4. Discussion

Motion dynamics investigations of occlusal and physical movement functions [22,23] have only described limb muscle output values during occlusal function exertion; studies analyzing the occlusal status (i.e., the mandibular position during muscle force exertion in detail) are lacking. Thus, in the current study, masticatory muscle activities and mandibular jaw movements during dynamic trunk muscle force exertion were simultaneously recorded and examined to address this knowledge gap in natural mandibular dynamics during muscle force exertion in humans. The results showed that the mandibular incisal point during dynamic trunk muscle force exertion was displaced from the maximal intercuspal position to a posteroinferior position, reflecting an open-mouth position. Additionally, in terms of masticatory muscle activities, the simultaneous activities of not only the Dm (responsible for mouth-opening) but also the Tm and Mm (responsible for mouth-closure) led to mandibular fixation.

Among the many exercises that exert trunk muscle force [24], the deadlift was adopted in the current study as an exercise with dynamic trunk muscle force exertion for the following reasons. First, the deadlift is a well-known powerlifting performance exercise, in which a barbell is lifted using mainly the back/buttocks/legs, including the latissimus dorsi, trapezius, erector spinae, gluteus maximus, and hamstring muscles, supporting the trunk. Second, as the deadlift is gradually implemented, and the upper body is used to keep the head to the back straight, the trunk posture is maintained, the head shakes less frequently, and jaw movements can be measured. Third, even participants with little experience in deadlifting can learn how to perform it safely and easily. For these reasons, the deadlift was selected as the test exercise.

It is of interest to determine whether the maxillary and mandibular teeth contact each other during physical exercise exertion. Some previous studies have investigated tooth contact by inserting a T-Scan sensor or a memory wafer into the mouth [25]. Although these methods are reliable for assessing tooth contact, the measurements are taken with participants holding a foreign body in their mouth during exercise exertion; thus, it is doubtful that the mandible is in a natural position. As an objective method for solving this problem, we had participants wear a jaw movement measuring instrument while attempting the test exercise. The ARCUS digma2, a six-degree-of-freedom jaw movement measurement instrument used in the current study, has the following properties: (1) it is lightweight and does not place a burden on the participant; (2) it is stable during assessment if an appropriate exercise is selected, like the deadlift; (3) it can measure natural jaw movements, as adequate clutch adjustment prevents the instrument from disturbing mandibular jaw movements; and (4) it can record mandibular jaw movements in real-time and show the three-dimensional trajectories of the incisal and condylar point movements on a computer screen resolution of 1/100th. Additionally, the only jaw movement measurement device available for this study was the ARCUS digma2. For these reasons, the participants in the current study wore this measurement device for assessment. The instrument was not displaced while the participants wore it, and stable measurements were achieved.

In the deadlift, which exerts dynamic trunk muscle force, the mandibular incisal point was displaced significantly posteriorly and inferiorly from the maximal intercuspal position of the sagittal plane to an open-mouth position during Pull, relative to Ready as the baseline position. Additionally, as the displacement was 0.24 ± 0.50 mm in the anterior–posterior direction and 0.55 ± 1.07 mm in the superior–inferior direction, the muscle force was exerted in a position posterior and inferior to the maximal intercuspal position (i.e., in a space not contacting any teeth) during Pull. During Down, the mandibular incisal point did not return to the maximal intercuspal position, and the position achieved during Pull was maintained. In the current study, mandibular jaw movements were observed as incisal point movement in the sagittal plane. As mandibular jaw movements are considered to reflect concerted movements based on Bonwill’s triangle (consisting of the incisal point and both condylar points), incisal point movements can be regarded as mandibular movements synchronized with condylar point movements [26]. Displacements from the maximal intercuspal position to a posterior position have been reported as likely to cause jaw dysfunction and have been reported from the viewpoint of abnormal stomatognathic function [27]. However, Sicher [28] reported that the working-side condyle during the Bennett movement not only rotates but also moves side-to-side, up-and-down, or inward and that the non-working-side condyle moves downward and forward. Additionally, some studies have reported that there is a space (0.5 to 1 mm) on the side at the rear of the joint cavity in which the working-side condyle can rotate during lateral movements and while backing away [29,30,31]. Moreover, Posselt reported on condylar point displacements during spontaneous backward movements and stated that, when the condylar point was placed posterior to the maximal intercuspal position, with tooth contact, the condylar point was displaced 0.3 mm posteriorly and 0.2 mm superiorly at the same order of magnitude as the changes observed in the current study [32]. Thus, the condyles can be displaced posteriorly because of the articular structure. In the current study, under trunk muscle force exertion, this space may enable fixation of the mandible, not in the maximal intercuspal position but in a posteroinferior position. Therefore, the hypothesis that the mandible is spontaneously displaced during strong muscle force exertion in dynamic movement is supported by the current study results.

It is also vital to know the activities of the masticatory muscles attached to the mandible when the mandible is displaced posteroinferiorly during muscle force exertion. From the viewpoint of the muscles involved in running, the posterior belly of the Tm and the Dm pulling the mandible posteriorly are considered to have significant effects on this type of event [33]. For inferior displacements, the suprahyoid and infrahyoid muscles are considered to contract and act in concert to fix the hyoid bone. By measuring jaw movements and masticatory muscle activities simultaneously, the current study showed that the Tm contraction increased during Pull and decreased during Down, to the same value as that during Ready and that the Mm and Dm contractions increased during Pull and decreased during Down. Based on these results, it can be stated that the masticatory muscles act to fix the displaced mandible and naturally support the results of the jaw movements. Asano et al. [34] examined masticatory muscle activities during maximum back muscle force exertion and reported Tm, Mm, and Dm contractions of 32.1%, 26.4%, and 97.4% of the MVC, respectively. Asano et al. also considered that, in light of the muscles involved in running, the jaw was displaced posteriorly because the mouth was not visually confirmed to be opened wide during measurements. This is consistent with the results of the current study, even though the percentage of the Dm contraction differed, which may be related to the fact that the barbell weight was approximately 80% of the maximum weight the participant could lift in the current study, while the maximum back muscle force exertion used in Asano et al. [34]. In any case, the hypothesis that the muscles responsible for mouth-opening and mouth-closure simultaneously contracted to fix the displaced jaw was supported.

The results of the current study are novel in that the contractions of the Tm, Dm, and Mm were approximately three times higher during Pull (when the maximum muscle force was exerted) than during Ready, even though the jaw was not placed in the maximal intercuspal position. In other words, Mm activity was observed under no occlusal contact. Only a few studies have investigated human jaw movements and masticatory muscle activities during natural muscle force exertion. In recent years, the concept of bracing has been introduced as firmly fixing the mandible in a position under no occlusal contact [35]. Bracing is currently defined as forcefully maintaining a certain mandibular position/activity without the necessary presence of tooth contact, or as an increased level of masticatory muscle activity without tooth contact [36]. Sugihara et al. reported that during a head-raising exercise, the Mm was activated even though the maxillary and mandibular teeth did not contact each other, which was considered to reflect bracing [37]. Bracing is considered to occur even during dynamic exercise [36], and the results of the current study suggest fixation of the mandible by bracing. For these reasons, under conditions where strong bracing is encountered, the dentist should consider movements beyond the previously established limitation in mandibular movement. Events that may be attributed to occlusion, such as tooth wear and/or bony prominence in athletes, may not be caused by clenching during exercise. Bracing should be taken into account during selection of a prosthetic device and occlusion adjustment. Future studies examining jaw function should also consider this factor. Nevertheless, the novelty of this study is highlighted, and we believe that it contributes to the literature in the field of sport dentistry as it provides important novel information for the readership.

The current study has several limitations to acknowledge. First, only the deadlift and relatively young adult males were evaluated; other exercises and various age groups should also be assessed in future studies. Moreover, information from mechanoreceptors in the periodontal ligament by mechanical stimuli to the teeth is known to affect masticatory muscle activities [38]. However, mandibular jaw movements without tooth contact are considered to be quite natural but unique, movements blocking information from the teeth by occlusal contact peculiar to the participant. Therefore, in the current study, mandibular jaw movements without occlusal contact during trunk muscle force exertion were considered to be mainly affected not by information from the teeth but by sensory information from the jaw joint, masticatory muscles, face, and skin mucosa around the mouth. It is necessary to examine this mechanism in future studies. Additionally, as mandibular jaw movements were observed two-dimensionally, using incisal point displacements in the sagittal plane, it is necessary to examine mandibular jaw movements three-dimensionally, including condylar point displacements. Moreover, it is considered necessary to examine the muscles involved in mouth-closure, such as the Tm and Mm, and muscles involved in mouth-opening, such as the Dm (which are suprahyoid muscles), along with the infrahyoid and neck muscles, such as the sternocleidomastoid and trapezius muscles, and to examine jaw movements during muscle force exertion in greater detail.

## 5. Conclusions

During dynamic trunk muscle force exertion, the mandibular incisal point was displaced from the maximal intercuspal position to a posteroinferior position, without tooth-touch (an open-mouth position). Additionally, in terms of masticatory muscle activities, the activities of not only the muscles involved in mouth-opening, such as the Dm, but also those of the muscles involved in mouth-closure, such as the Tm and Mm, led to mandibular fixation, which is considered to reflect a type of mandible fixing known as bracing.

## Figures and Tables

**Table 1:** Jaw movements during the steps of the deadlift (Ready, Pull, Down).

Incisal Movement in the Sagittal Plane	Ready (Reference)		Pull (mm)		Down (mm)		F	*p*	Multiple Comparisons
	M *	SD *	M	SD	M	SD			
*X*-axis	-	-	−0.24	0.50	−0.17	0.35	4.12	0.023	Ready > Pull, Ready > Down
*Y*-axis	-	-	−0.55	1.07	−0.30	0.97	4.30	0.019	Ready > Pull

*: M: mean; SD: standard deviation.

**Table 2:** Muscle activities during the steps of the deadlift (Ready, Pull, Down).

	Ready (% MVC)		Pull (% MVC)		Down (% MVC)		F	*p*	Multiple Comparisons
	M *	SD *	M	SD	M	SD			
Temporalis muscle	6.90	4.19	18.63	17.17	10.39	12.11	11.44	<0.001	Pull > Ready, Down > Ready
Masseter muscle	7.06	5.00	21.21	18.73	11.79	11.94	18.13	<0.001	Pull > Down > Ready
Digastric muscle	6.98	4.25	21.82	19.97	13.82	14.43	15.56	<0.001	Pull > Down > Ready

*: M: mean; SD: standard deviation; MVC: maximal voluntary contraction.
